# Influences of scan-position on clinical ultra-high-resolution CT scanning: a preliminary study

**DOI:** 10.1038/s41598-018-37514-6

**Published:** 2019-02-04

**Authors:** Lu Li, HuiMin Li, JinEr Shu, JiangFeng Pan, XiaoRong Chen, MingLiang Ying, YiBin Xu, Dingjun Wang, Peipei Pang

**Affiliations:** 1 Department of Radiology, Jinhua Central Hospital of Zhejiang University, Jinhua, 321000 China; 2 Department of Radiology, Xinhua Hospital Affiliated to Shanghai Jiaotong University School of Medicine, Shanghai, 200092 China; 3 GE healthcare, Hangzhou, 310000 China

**Keywords:** Cancer imaging, Cancer imaging, Cancer, Cancer, Clinical trials

## Abstract

The aim of this study is to access influences of scan-position on clinical ultra-high-resolution CT scanning. We proposed a breath-hold assisted ultra-high-resolution scanning technology (scan scheme G) and compared with scan scheme A (regular CT plain scan) and scheme B (1024 ultra-high-resolution scan with patients stay in supine position). A total of 30 patients with fGGO were included in this study. Three highly experienced chest imaging doctors were employed to score the image and to select regions of interest (ROIs) for CT value and signal-to-noise ratio (SNR) calculation. In comparison with scan A and B, this new scan scheme G shows more clear CT images and higher SNRs at overall lung field (the p-values of A versus G and B versus G are 0.041 and 0.065, respectively). These findings suggest that scan-G provides a better image quality and contributes significantly to clinical detection accuracy of fGGO.

## Introduction

The localized pulmonary focal ground glass opacity (fGGO) is a key way to diagnosis of the early lung cancer, thus, analyzing the characteristic of fGGO is quite important^1–5^. With the widespread use of low-dose spiral CT (LDCT), the case detection rate of fGGO has significantly increased^6–8^. However, how to correctly find and handle the fGGO is still the hotspot and difficult issue for imaging workers^9–11^, and patients may require a repeated follow-up CT examination if the nodules cannot be clearly diagnosed. The development of Multi-Detector Spiral CT (MDCT) provides higher imaging resolution, which employs target scanning and large matrix acquisition to get smaller pixels^12–15^. The identifiability of initial characteristic and boundary of nodules may be highly improved by taking this ultra-high-resolution technique and breath-hold^16^. In this study, we proposed a new CT scanning scheme G, namely the breath-hold assisted ultra-high-resolution scanning technology, to improve the imaging quality of fGGO diagnosis. The imaging technique was clinically tested with 30 consecutive cases. The patients were scanned with 3 different CT protocols, and we compared the imaging quality. Both the CT images and statistical analysis show a significant improvement in image quality, indicating the important clinical application of the G-scan protocol for nodule diagnosis.

## Materials and Methods

### Patients

All procedures involving human participants were in accordance with the ethical standards of the ethical committee of Jinhua Central Hospital of Zhejiang University and with the 1964 Helsinki declaration and its later amendments or comparable ethical standards. All of the patients signed the informed consent to the scientific use of their information or bio-specimen before surgery.

The study is conducted on 30 consecutive cases of followed-up (3 to 12 months) fGGO patients during routine low-dose lung screening, which is comprised of 9 male and 21 female; The age vary from 35 to 74 with the average of 50. CT scans are performed on the region of nodules that were detected. The CT scans contain three schemes: regular CT plain scan (retrospective 1024 matrix target reconstruction (scan scheme A)), 1024 ultra-high-resolution scan (scan scheme B), and breath-hold assisted ultra-high-resolution scan (scan scheme G).

### CT Scanning Protocol

All CT scanning protocols were approved by the ethical committee of Jinhua Central Hospital of Zhejiang University. All CT scans adopt Philips iCT 256 machine model (Brilliance iCT, Philips, Holland). The reconstruction matrix size of iDose^4^ (iterative reconstruction IR, level 4) is 512 × 512 mm and pulmonary window thickness is 1 mm with an interval of 1 mm. To avoid motion artifacts and get the best imaging quality, patients are trained to breathe before CT scanning and CT images are obtained when patients are breath holding after taking a deep breath. The scanning range is centered on the nodule and covered 3-cm length at the top and bottom respectively. All the scans adopt the helical scanning (0.625 mm × 128).

For scan scheme A, the scanning method is the regular plain scan (the FOV is 500 mm with a scan matrix of 512 × 512), patients lie in supine position and repeat the action of “breath-in-and-hold-breath”. The reconstruction slice thickness is 5 mm with an interval of 5 mm; The voltage and current are 120 KV and 40–100 mAs respectively. FBP-D algorithm (C-500HU W 1500HU) is used to reconstruct pulmonary window. The scan time is 1.395 s, and the average CTDIvol is 5 mGy and the DLP is 87 mGy*cm with a minimum scan range of 8 cm. Raw data from regular plain scan (no need for re-scanning) is used for retrospective target reconstruction. The FOV is 150–215 mm to display nodule and position structure. The thickness is 0.67 mm with an interval of 0.67 mm and the matrix size of iDose^4^ is 1024 × 1024 mm. All images are smoothed with lowpass filter(A).

For scan scheme B, the scanning method is 1024 ultra-high-resolution scan (the FOV is 250 mm with a scan matrix of 1024 × 1024). Patients stay in supine position and the scan voltage and current are 120 KV and 250 mAs; The scan time is 3.917 s, and the average CTDIvol is 22.12 mGy and the DLP is 174.6 mGy*cm with a minimum scan range of 6 cm; The reconstructed FOV is 149–215 mm and thickness is 0.67 mm with an interval of 0.67 mm; iDose^4^ is used and images are smoothed with lowpass filter(A).

For scan scheme G, 1024 ultra-high-resolution scan (the FOV is 250 mm with a scan matrix of 1024 × 1024) is employed. Patients lie in lateral or slanting lateral position to make the nodule above the scanning lung field (to make the nodule and its background inflate/over-inflate by respiratory physiology). The scan view angle is 90° and the scan voltage and current are 120 KV and 250 mAs; The scan time is 3.917 s, and the average CTDIvol is 22.12 mGy and the DLP is 174.6 mGy*cm with a minimum scan range of 6 cm; The reconstructed FOV is 150–215 mm and thickness is 0.67 mm with an interval of 0.67 mm; iDose^4^ is used and images are smoothed with lowpass filter(A).

### Evaluation method

All CT images are transferred to the workstation of PHILIPS EBW V4.5.5 for online processing. In order to make sure the CT images are clear enough for nodule diagnosis, three highly experienced chest imaging doctors are employed to evaluate the images from the subjective and objective perspective.

The subjective evaluation of image quality is based on the details of ground-glass opacity (GGO). The anonymous CT images of the same nodule were shown side by side in a random manner and doctors observe the contour and internal density structure of the GGO, including the distribution and clarity of background small blood vessels of the lung. When they viewed the CT images, the readers were asked to make a diagnosis, and evaluate the quality of CT findings and grade the images according to their helpfulness for diagnosis using a 5-point score as follows:

Each CT finding, including the lobulation sign, spiculation, pleural indentation, bubble sign, margin of nodule, solid component was subjectively evaluated and graded using a 5-point score according to a previous report^17^: ‘1’ indicated worst image quality which means no detectable findings; ‘2’ indicated poor image quality that findings can be detected but the margin or internal characteristics are difficult to evaluate; ‘3’ indicated fair image quality in which partially indistinct findings can be detected and the margin or internal characteristics can be evaluated; ‘4’ indicated good image quality in which some indistinct findings can be detected and the margin or internal characteristics can be evaluated; and ‘5’ indicated excellent image quality in which findings are extremely clear and easy to detect, and the margin or internal characteristics can be evaluated.

The typical CT value of GGO is used to compare the image quality objectively. We choose five 40-mm^2^ ROIs (inner, center, outer, former, posteriori) of the lung fields on the fGGO layer for CT value calculation.

### Statistical analysis

A P value of less than 0.05 was considered statistically significant. Comparisons of the SNR are also listed. SNR is the log compression of the ratio between signal power and background power within ROI regions, which is shown in the bellow Eq. (1):

1 $$SNR(dB)=20\,\times \,{\mathrm{log}}_{10}(\frac{Power\_signal}{Power\_background})$$

where ${Power\_signal}$ and ${Power\_background}$ denote the signal power and noise power in the region of interest, respectively.

## Results

As a result, 30 patients with fGGO were included in the final analysis. Of the 30 nodules, 21 were pure ground glass nodules, and the remaining 9 were mixed ground glass nodules. The size of the 30 nodules ranged from 6 mm to 22 mm.

### CT Image comparison

**Figure 1:**
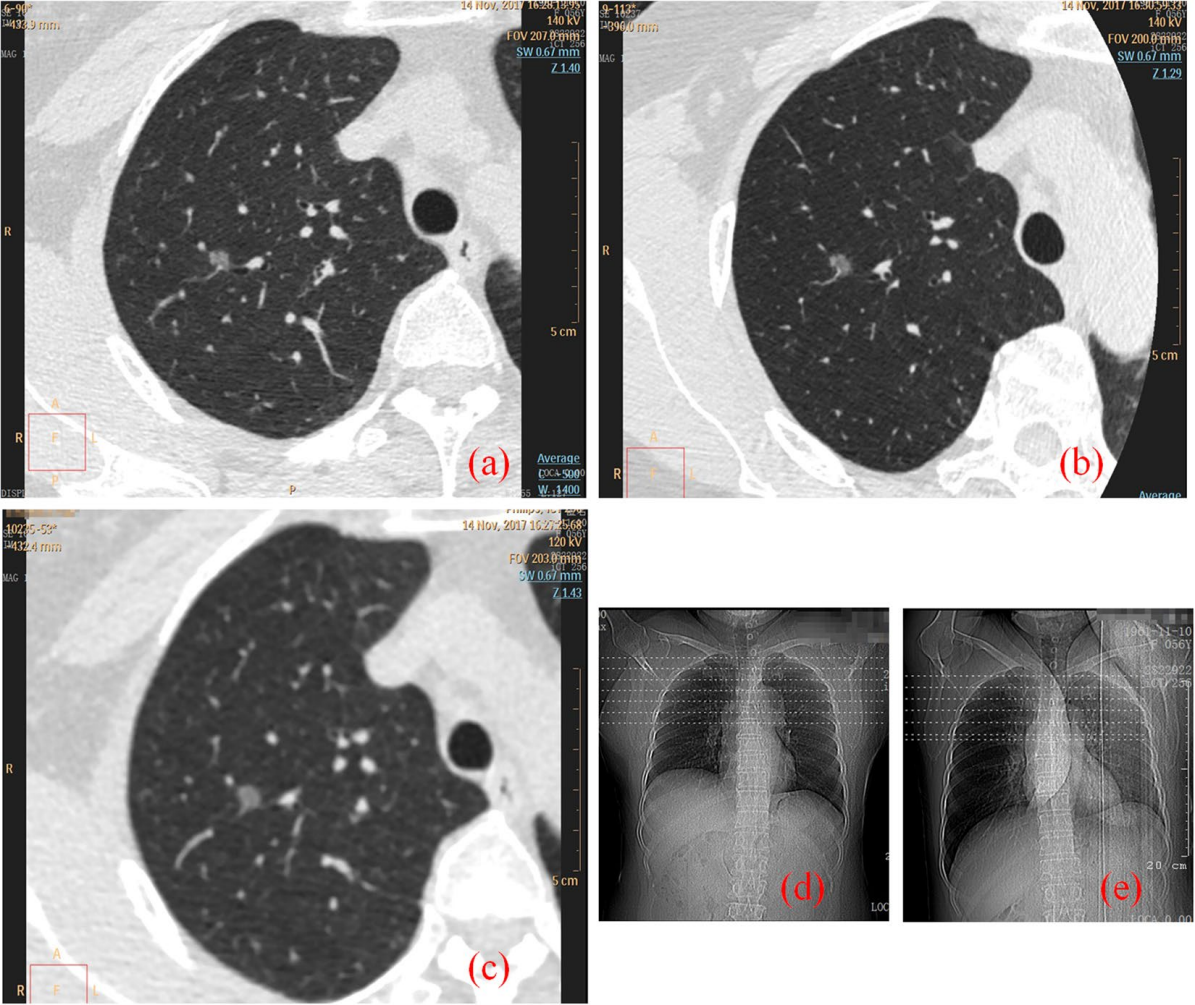
Comparison of nodule cross-sectional imaging for the three CT scanning schemes. The nodule was located in the lung. The images were provided by department of radiology of Jinhua Central Hospital to Zhejiang University and were from a 57-year-old female patient. (**a,d**) 1024 ultra-high-resolution scan with patients stay in supine position (B-Scan). (**b,e**) Breath-hold assisted ultra-high-resolution scan with patients lie in lateral position (G-Scan). (**c,d**) Retrospective 1024 matrix target scan with patients stay in supine position (A-Scan).

Figure 1 shows cross-sectional images of two representative cases with three CT scanning schemes. The nodule is located in the lung, and ROIs are chosen for statistical analysis. The edge of nodules and solid internal components were shown more clearly on the G-scan image than either on the B-scan image or A-scan image.

**Figure 2:**
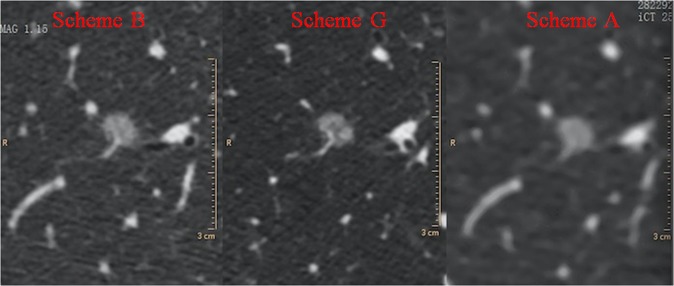
Local enlarged nodule images for the three CT scanning schemes. Scan scheme G improves spatial resolution and provides better detail detections of nodule structure.

Figure 2 is comparison of local enlarged nodule images for the three CT scanning schemes. Scan scheme G improves spatial resolution and provides better detail detections of nodule structure.

### Objective Evaluation

**Figure 3:**
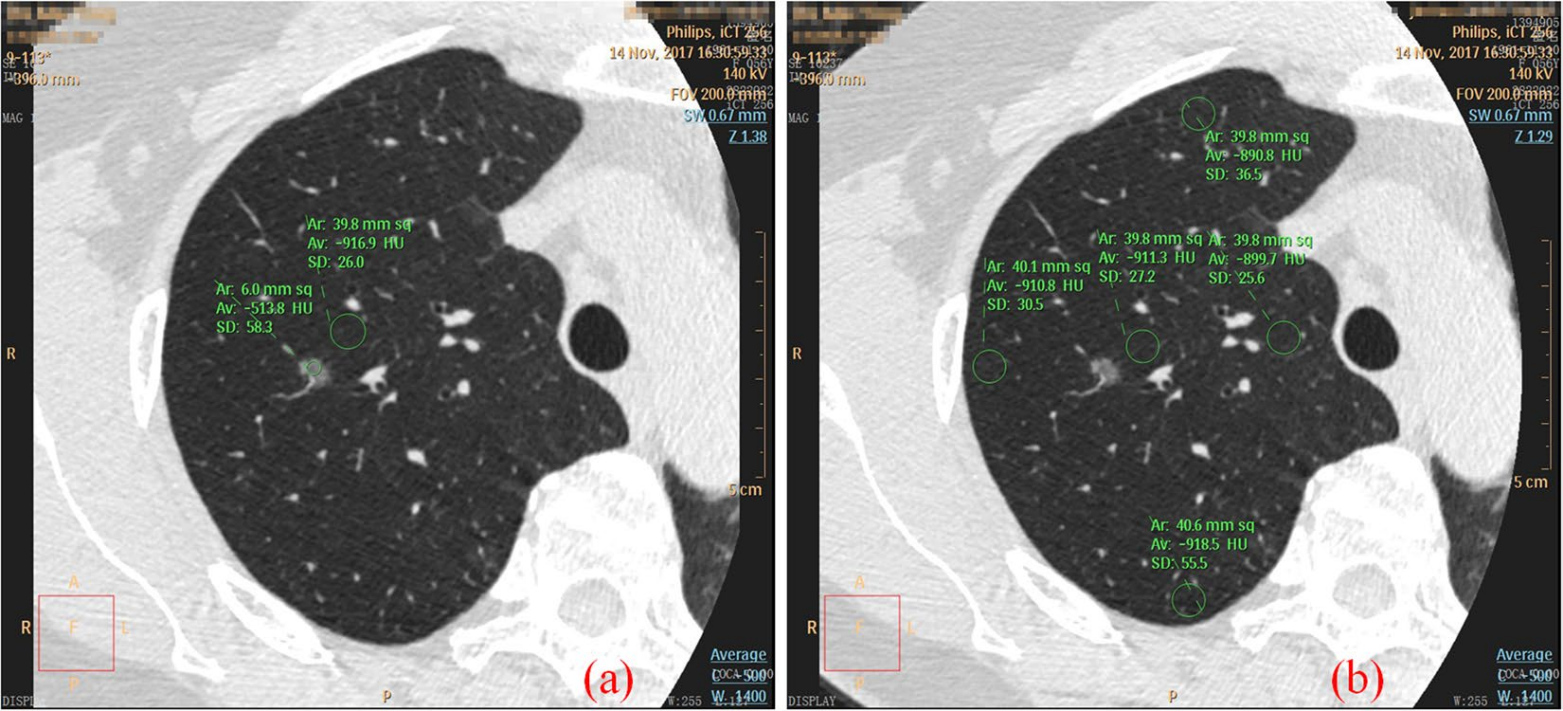
Illustration of ROIs. (**a**) ROI location for SNR calculation; (**b**). ROI location for CT value calculation: inner ROI, center ROI, outer ROI, former ROI and posteriori ROI.

Comparison of imaging scores for the three CT scanning schemes was shown in Table 1. Illustration of ROIs (inner, center, outer, former, posteriori) of the lung fields on the fGGO layer for CT value calculation was shown in Fig. 3(b). The image quality of the scheme G findings has significantly higher score than that of the B-scan and A-scan regarding the margin and internal characteristics (all P < 0.05).

**Table 1:** Comparison of imaging scores for the three CT scanning schemes.

Findings	Scan-A		Scan-B		Scan-G		p-value			Samples
	M	SD	M	SD	M	SD	p1	p2	p3	
Margin of nodule	3.17	0.38	3.80	0.41	4.87	0.35	0.000	0.000	0.000	30
Margin of solid component	3.00	0.47	3.90	0.32	4.90	0.32	0.000	0.000	0.000	10
Lobulation sign	3.59	0.51	3.95	0.23	4.95	0.23	0.002	0.000	0.000	19
Pleural indentation	3.33	0.50	3.67	0.30	4.89	0.33	0.176	0.000	0.000	9
Bubble sign	3.33	0.38	3.86	0.38	5.00	0.00	0.004	0.000	0.000	6
Air bronchogram	2.75	0.45	4.20	0.45	5.00	0.00	0.000	0.000	0.000	5
Margin of nodule blood vessel	3.05	0.21	3.91	0.29	4.91	0.29	0.000	0.000	0.000	22

Note—P values were calculated with independent sample t test, the p-value of less than 0.05 was considered to indicate a statistically significant difference. P1 indicates the P values for t-test of A schemes versus B schemes; P2 indicates the P values for t-test of A schemes versus G schemes; P3 indicates the P values for t-test of B schemes versus G schemes. M: Mean value; SD: Standard Deviation.

### Subjective evaluation

**Figure 4:**
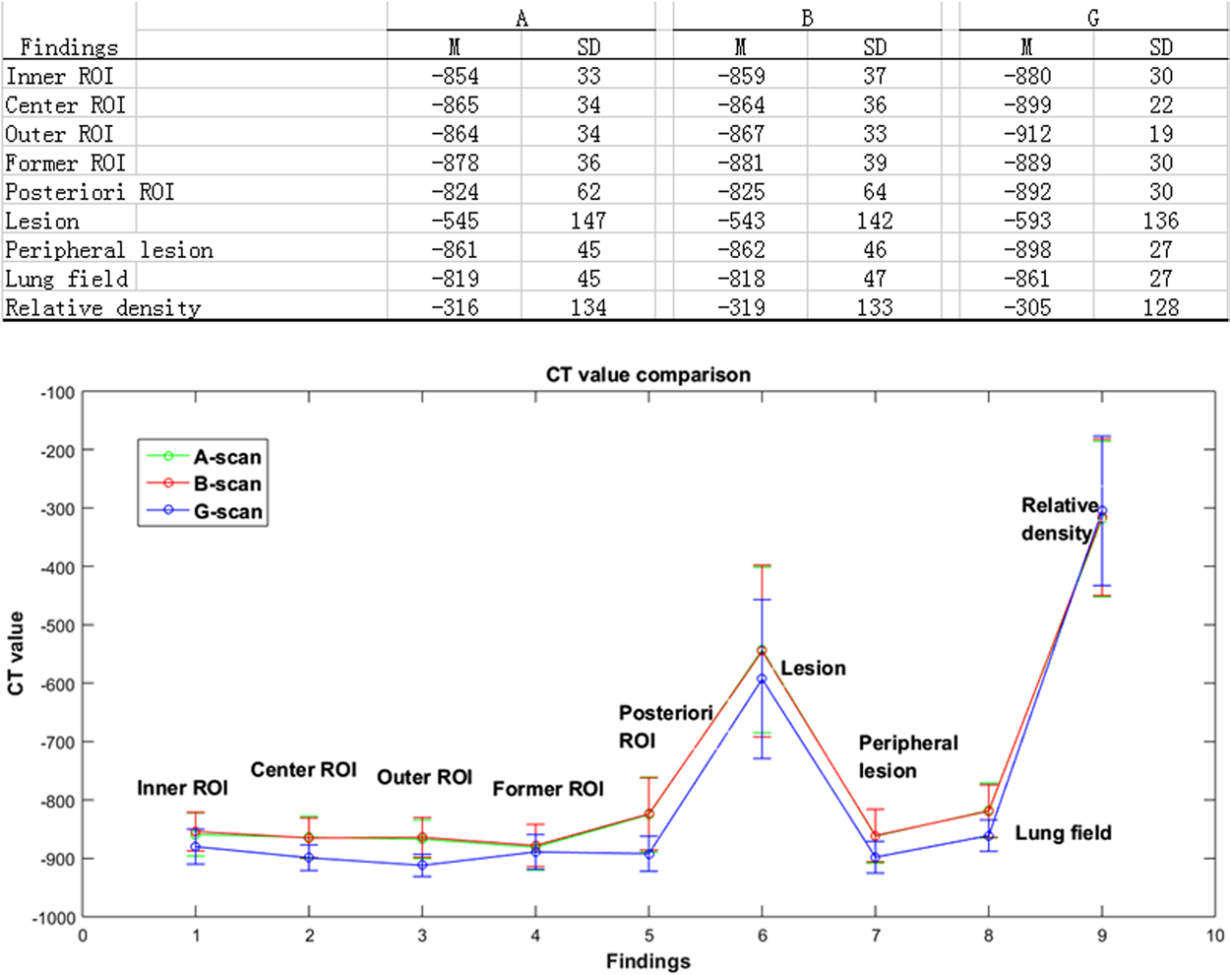
Comparison of CT values for the three CT scanning schemes.

Figure 4 compares the density gradient (CT value) of the CT images of the three scan methods. Referring to the line chart in Fig. 4, scan-G shows lower CT values than scan-A and scan-B, which directly proves that the imaging resolution has been significantly improved with scan scheme G.

**Figure 5:**
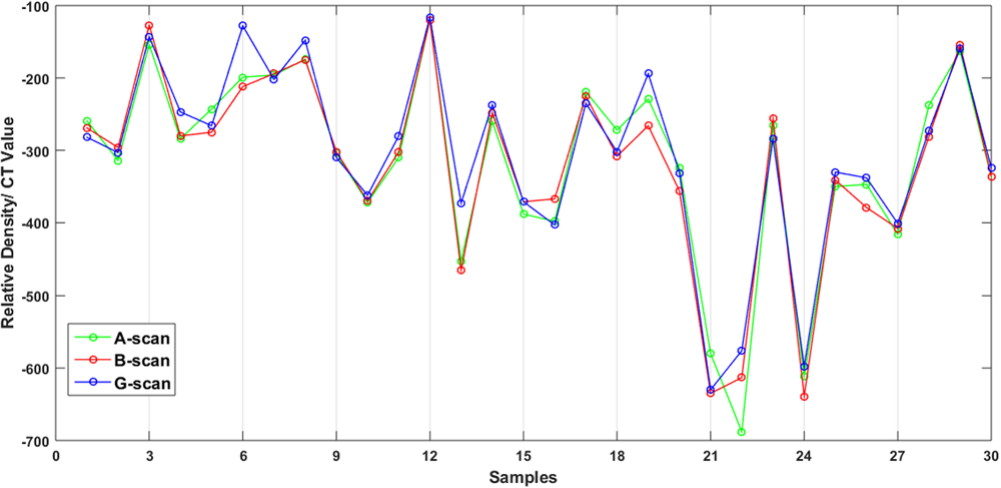
Relative density values for the three CT scanning schemes.

Figure 5 is the relative density comparison and relative density does not change much for this all scan schemes statistically.

### Quantification assessment

**Figure 6:**
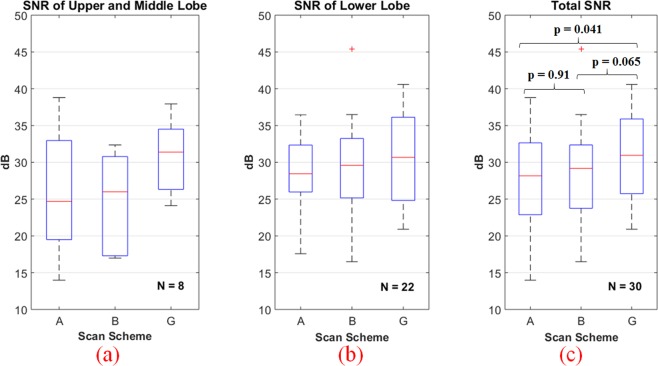
Box-and-whisker plot of SNRs of different lung field for the three CT scanning schemes. (**a**) SNR comparison of upper and middle lobe, N = 8; (**b**) SNR comparison of lower lobe, N = 22; (**c**) Total SNR comparison of 30 samples. The p-values of A versus G, B versus G, and A versus B are 0.041, 0.065, 0.91, respectively.

Doctors selected ROIs of fGGO for SNR calculation (Fig. 3(a)). We analyzed the SNR values of different lung field (upper, middle and lower lobe) for the three CT scanning schemes., which are shown in Fig. 6(a–c) shows the total SNR comparison of 30 samples. For scan scheme A and B, SNR of upper and middle lobe are higher than lower lobe, while the SNRs of scan G are similar high at each lung field, also SNRs of scan G are overall higher than scan A and B. The p-values of A versus G, B versus G, and A versus B are 0.041, 0.065, 0.91, respectively.

**Figure 7:**
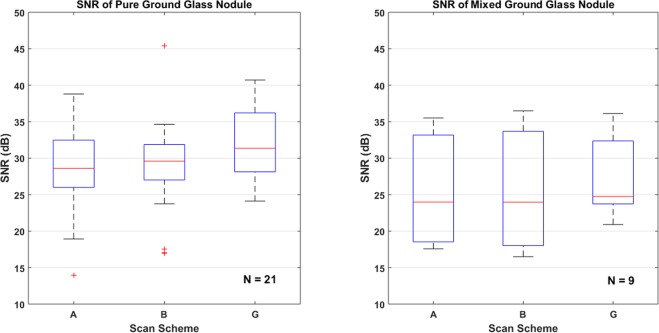
SNR value comparisons of different type of ground glass nodule for the three CT scanning schemes.

Figure 7 is the SNR value comparisons of different types of ground glass nodule for the three CT scanning schemes. For scan scheme A, B and G, SNRs of pure ground glass nodule are higher than mixed ground glass nodule. In the other hand, scan-G provides better imaging quality than scan A and B.

## Discussion

Accurately demonstrating the minutia of lung lesion is the key to diagnosing small pulmonary nodule, which raises demand for higher spatial resolution and contrast of imaging technique^17–20^. The confirmed diagnosis of small pulmonary nodule, especially those with fGGO, is closely related with the characteristics of inner structures containing uniformity and distribution feature, edge characteristic, and correlation of the background lung structure^21^. The only way to fully reveal the above-mentioned features is to reduce the imaging pixel as small as possible, namely, to increase the spatial resolution. The most frequently used resolution improvement method named “ultra-high-resolution target scanning” includes narrowing scanning field of view (target scanning), enlarge scanning matrix (1024 matrix), and ultrathin layer thickness (submillimeter), yet low-dose is rarely employed for reducing imaging noise^5–9^. With the development of MDCT device, this “ultra-high-resolution target scanning” that has 1024 × 1024 scan matrix, 250 mm FOV and 0.24 mm pixel, considerably raises spatial resolution in comparison with the routine low-dose scan (0.98 mm pixel). However, noise may be obviously amplified as the pixel is smaller, thus, it hinders the further increase of resolution and even decreases resolution^22^. Therefore, it is quite necessary to adopt iterative algorithm like iDose^4^ to lower image noise to keep high imaging quality.

Definition of feature details of small pulmonary nodule, particularly the fGGO, depends a lot on inflation degree of background lung tissue^23,24^; When the lung inflates fully (CT value decrease), the radiolucency and definition of lung filed will increase, and the lung vessels are fully stretched; as a result, nodule and nodule-lung interface will be effectively showed. Furthermore, hyperinflation other than simple deep inspiration would be more helpful for the nodule display. In this study, taking advantage of scan position and breath-hold, we propose a new ultra-high-resolution CT scan method (scan scheme G), which scans the nodule above the scanning lung field with patients lying in lateral or slanting lateral position. We compare this new scan scheme G with scan scheme A and B regarding image quality and analysis is based on whether it has a comparative advantage on the resulting diagnosis. To our knowledge, this prospective clinical study is the first evaluating the image quality and diagnosis accuracy for the detection of pulmonary nodules using scan scheme G compared to that of A and B. This technique is realized by combining breath-holding of patients with traditional ultra-high-resolution scanning technique. This combination cost-effectively improves the imaging quality and provides detail detections of fGGO, indicating its important clinical application for helping doctors to better diagnose fGGO. Moreover, during our experiment, scan scheme-G could better detect both the non-fGGO type nodule and the fGGO type nodule than scan scheme-B.

Except for improvement of the imaging quality, we also find out that there is no obviously different in relative density of these three scan schemes statistically. However, more evidence need to be provided. Therefore, further study should be performed to determine whether relative density is a reliable parameter for evaluating fGGO.

By combining this breath-hold assisted ultra-high-resolution scanning technology with target reconstruction, we can further get more information about the feature of nodule from various perspectives, which may bring higher case detection rate of fGGO. Briefly, this combination includes two imaging reconstructions: one is the high spatial resolution data with 1–2 mm layer for detecting minutiae of nodule feature; the other is the low spatial resolution data with 0.67 mm layer for density analysis and three-dimensional reconstruction such as MIP, minIP and VR.

To summarize, breath-hold assisted ultra-high-resolution scanning technology not only improves spatial resolution, but also provides detail detections of nodule structure, especially for fGGO. In comparison with scan scheme A and scheme B, this new scan scheme G shows great clinical use for fGGO detection and indicates better detection accuracy.

## Conclusion

This study is the first to combine scan position and breath-hold during ultra-high-resolution CT scan for fGGO diagnosis. The imaging technique was clinically tested and compared with other scan protocols. This new method of ultra-high-resolution CT scanning provides a significantly better image quality and can be clinically used to evaluate lung nodule.
