# Author Correction: Influences of scan-position on clinical ultra-high-resolution CT scanning: a preliminary study

**DOI:** 10.1038/s41598-019-44356-3

**Published:** 2019-05-22

**Authors:** Lu Li, HuiMin Li, JinEr Shu, JiangFeng Pan, XiaoRong Chen, MingLiang Ying, YiBin Xu, Dingjun Wang, Peipei Pang

**Affiliations:** 1 Department of Radiology, Jinhua Central Hospital of Zhejiang University, Jinhua, 321000 China; 2 Department of Radiology, Xinhua Hospital Affiliated to Shanghai Jiaotong University School of Medicine, Shanghai, 200092 China; 3 GE healthcare, Hangzhou, 310000 China

Correction to: *Scientific Reports* 10.1038/s41598-018-37514-6, published online 04 February 2019

The Acknowledgements section in this Article was omitted. The Acknowledgements section should read:

“This work was supported by Zhejiang Provincial Natural Science Foundation (Grant Number LGF18H180011) and Jinhua Science and Technology Bureau (Grant Number 2017-3-004)”.

